# Evaluation of the Thermal Stability of a Vaccine Prototype Based on Virus-like Particle Formulated HIV-1 Envelope

**DOI:** 10.3390/vaccines10040484

**Published:** 2022-03-22

**Authors:** Diana Aguado-Garcia, Alex Olvera, Christian Brander, Victor Sanchez-Merino, Eloisa Yuste

**Affiliations:** 1 Institute of Health Carlos III (ISCIII), National Microbiology Centre, 28220 Madrid, Spain; diaguadog@gmail.com (D.A.-G.); vsancmer@uax.es (V.S.-M.); 2 IrsiCaixa, AIDS Research Institute, Hospital Germans Trias i Pujol, 08916 Badalona, Spain; aolvera@irsicaixa.es (A.O.); cbrander@irsicaixa.es (C.B.); 3 Faculty of Medicine, Universitat de Vic-Universitat Central de Catalunya (UVic-UCC), 08500 Vic, Spain; 4 Centro de Investigación Biomédica en Red de Enfermedades Infecciosas (CIBERINFEC), 28029 Madrid, Spain; 5 Institució Catalana de Recerca i Estudis Avançats (ICREA), 08010 Barcelona, Spain; 6 Faculty of Health Sciences, Alfonso X el Sabio University, 28691 Madrid, Spain

**Keywords:** virus-like particles, HIV, vaccine, thermostability

## Abstract

The long-term storage stability of vaccines has a major impact on the roll-out and success of global immunization programs. For the Human Immunodeficiency Virus type 1 (HIV-1) virus-like particle (VLP) vaccine prototype evaluated here, nanoparticle tracking analysis (NTA), and enzyme-linked immunoabsorbent assay (ELISA) results demonstrated a remarkable structural stability. VLPs maintained their integrity and the recognition of relevant B-cell epitopes for three months at 4 and −20 °C. Interestingly, most particles remained intact and preserved the recognition of relevant epitopes even after a week of storage at room temperature.

## 1. Introduction

Maintaining the cold chain for the transport and storage of vaccines can be a major challenge in many regions of the world due to limitations in adequate storage infrastructure and guaranteed electricity access and lack of adequate storage places. In the case of a Human Immunodeficiency Virus (HIV) vaccine, this obstacle is especially limiting since the regions most affected by the pandemic are also the most impoverished. For this reason, the development of HIV vaccine prototypes that maintain their immunogenic capacity at ambient storage and transport temperatures is of special interest.

Vaccine prototypes based on HIV-1 envelope proteins incorporated into HIV Gag virus-like particles (VLPs) constitute promising vaccine prototypes, and have been shown to induce strong heterologous anti-gp120 responses [1,2]. Our group has developed several of these prototypes with an HIV-1 Gag sequence previously optimized as a T cell immunogen (dGag) and various different HIV-1 envelope sequences. The dGag sequence is based on the 2001 clade B Gag consensus sequence with modifications in 32 amino acid positions relative to the consensus sequence. These modifications were introduced to cover positions that are frequent targets of the virus-specific CD8 T cell response and include residues with more than 10% frequency in clade B sequences [3,4]. These VLP prototypes have previously been shown to induce potent heterologous antibody responses in rabbits [4,5]. However, their efficacy may be hampered if their conformation is not kept stable under readily achievable storage and transport conditions.

In this study, we evaluated the thermostability of such a VLP vaccine prototype by assessing particle integrity and viral spikes antigenicity, focusing on the functionality of the epitopes associated with the induction of broadly neutralizing antibodies (bnAbs), under both long-term and accelerated storage conditions.

Thermostability of the VLP formulated HIV-1 envelope protein at specific temperatures was assessed in two ways: One assessing particles integrity by nanoparticle tracking analysis (NTA) and the other by assessing the integrity of certain epitopes that are relevant to the induction of broadly neutralizing responses by enzyme-linked immunoabsorbent assay (ELISA).

## 2. Materials and Methods

### 2.1. Virus-Like Particle Generation

The envelope selected for VLP generation in this study corresponds to AC10_29 virus, which is a subtype B, fiebig 3, and tier 2 isolate (https://www.hiv.lanl.gov/content/immunology, accessed on 19 March 1998). No sequence modifications were introduced to the AC10 envelope sequence.

HIV-1-Gag VLPs were produced by transient transfection with the FreeStyle 293 Expression System (Invitrogen, Carlsbad, CA, USA) co-transfecting with a pcDNA-dGag plasmid, a furin expression plasmid, and a codon-optimized pcDNA3.1-AC10 envelope expression plasmid provided by GeneArt Gene Synthesis (ThermoFisher Scientific, Waltham, MA, USA). For transfection, 1 μg DNA/10^6^ cells of each dGag, Env, and furin expression plasmids were used at an 11.5:1:0.3 molecular ratio. At 48 h post-transfection, VLPs were harvested by ultracentrifugation. First, VLP supernatants were clarified through two rounds of centrifugation at 800× *g* for 5 min. Afterwards, the supernatants were ultracentrifuged at 50,000× *g* for 30 min. Pellets were resuspended in PBS, ultracentrifuged at 160,000× *g* for 10 min, and resuspended in trehalose (Sigma-Aldrich, St. Louis, MO, USA) 15%/PBS to preserve VLP integrity and stored at room temperature, 4 and −20 °C [6].

### 2.2. Nanoparticle Tracking Analysis (NTA)

Size distribution and particle concentration were analyzed by measuring the rate of Brownian motion using a Nanosight NS300 (Malvern Instruments, Malvern, United Kindom). Samples were serially diluted in particle-free PBS to reach a suitable particle concentration (60–100 particles per video frame) for analysis. Five videos at flow mode (60 s) of a 1/100 dilution for each sample were captured at room temperature. Videos were analyzed and evaluated by Nanosight NS300 NTA software. Camera level was adjusted manually, and optimized analysis parameters were kept constant during all measurements. Particle number was evaluated for particles with diameters between 10 and 1000 nm.

### 2.3. gp120 and VLP ELISAs

ELISAs with 447-52D antibodies and a monomeric recombinant gp120 (HIV-1 Bal) standard curve were used to quantify the amount of gp120 VLP incorporated. 447-52D was selected for quantification because it is specific to an exposed, conserved linear epitope. We used 50 µL of recombinant gp120 and VLPs serial dilutions in PBS to coat ELISA plates (Immulon, ThermoFisher Scientific, Waltham, MA, USA) and incubated at 4 °C overnight. Next, wells were blocked with 100 μL of 5% nonfat powdered milk in PBS at 37 °C for 1 h. 50 μL of 447-52D antibody (5 µg/mL) in 5% PBS-milk were added to each well and incubated at 37 °C for 1 h. After washing three times with PBS plus 0.05% Tween 20, 50 μL of horseradish peroxidase-conjugated goat anti-rabbit immunoglobulin G antibody (sc-2004, Santa Cruz Biotechnology, Santa Cruz, CA, USA) diluted 1/2000 in 5% milk in PBS was added to each well, and the plates were incubated at 37 °C for 1 h. Plates were then washed 6 times with PBS-Tween and then 50 μL of tetramethylbenzidine reagent (Calbiochem, San Diego, CA, USA) was added to each well. Five minutes later, 50 μL of hydrochloric acid was added to each well and optical density at 450 nm was measured using a spectrophotometer (Sunrise, TECAN, Zürich, Switzerland). For VLP ELISA, 50 µL of VLPs per well at 0.1 µg of gp120/mL were used to coat ELISA plates. MAb binding was then assessed by conducting an ELISA as described above, omitting detergent from PBS wash buffers [7].

To calculate residual binding percentages for each antibody, storage at 4 °C for 48 h were considered the initial conditions. For each storage temperature, antibody, and antibody concentration, the percentage of the initial optical density was calculated. Statistical analysis was performed using one-way analysis of variance (ANOVA). Differences in binding percentages between each storage condition and the corresponding initials were considered significant when *p* ≤ 0.05.

## 3. Results and Discussion

### 3.1. Particle Size Distribution

**Figure 1:**
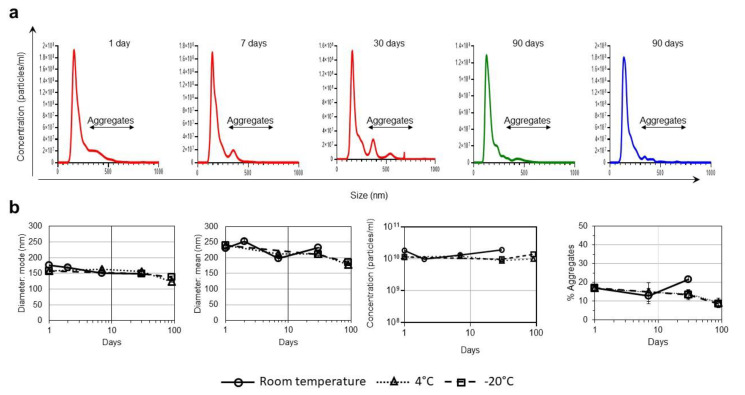
Effect of different storage temperatures in VLPs size and integrity. (**a**) Particle sizes and population distribution after 1, 7, and 30 days of storage at room temperature (red line); 90 days of storage at 4 °C (green line) and 90 days of storage at -20 °C (blue line) monitored by NTA. Black arrows identify the population of aggregates >300 nm. (**b**) Particle diameters (average mode and mean values with the corresponding standard deviations), particle concentrations, and percentage of aggregates over time after storage at room temperature, 4 and −20 °C, monitored by NTA. Values are averages and standard deviations of the values obtained in the five videos.

We found that the VLPs maintained a stable average diameter of 150 nm for three months at 4 and −20 °C (Figure 1). After one week at room temperature (24 °C), some aggregates of >300 nm in diameter were observed (12.97% of all particles), indicating the beginning of VLP degradation (Figure 1). However, 87.03% of all VLPs maintained structural integrity after one week of storage at room temperature and this number was only slightly reduced to 78.24% after a month of storage. In VLPs stored at 4 and −20 °C, the percentage of aggregates observed after three months of storage was similar to that of the original stock (Figure 1b).

In previous studies, we used electron cryo-microscopy techniques for VLP characterization and identified a pseudovirus-like structure that can be attributed to the presence of HIV-Gag and VLP sizes very similar to those obtained by NTA [4]. The present study has been possible thanks to the use of NTA for particle size analysis of millions of particles, which is not feasible by electron microscopy approaches.

### 3.2. Comparative Antigenicity

**Figure 2:**
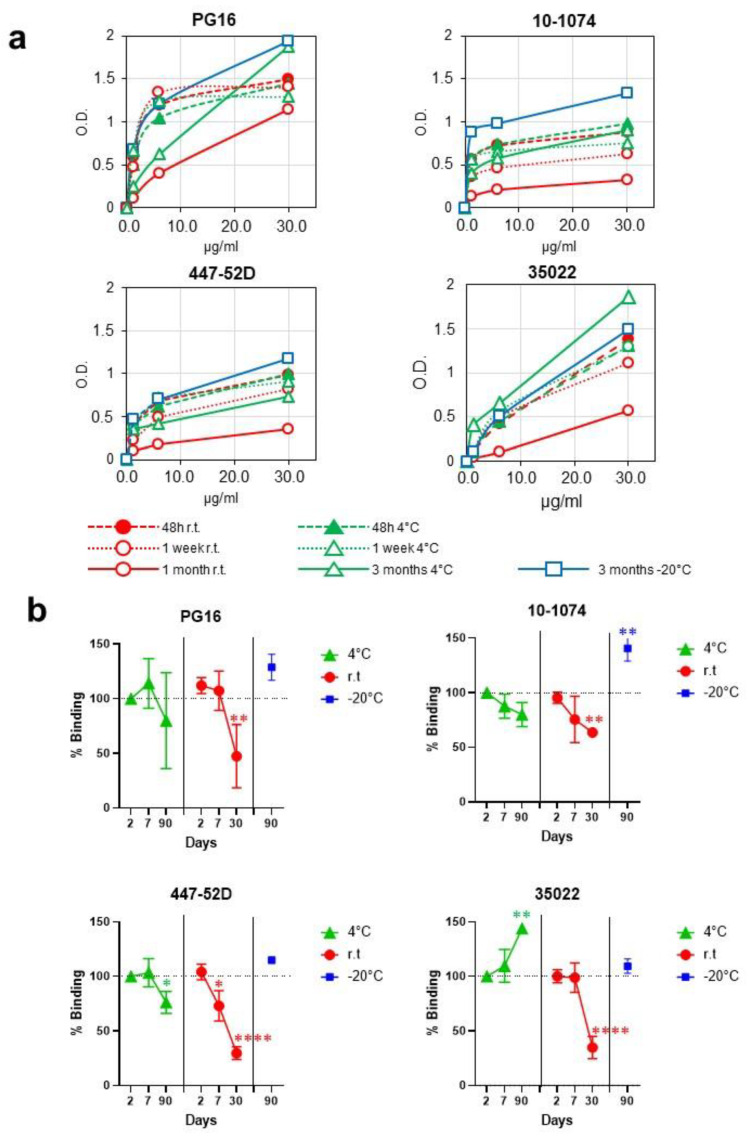
B-cell epitope preservation after storage at different temperatures. (**a**) Antigenicity of VLPs formulated Envs was evaluated by ELISA with a panel of monoclonal antibodies with different specificities. VLPs were normalized by gp120 and immobilized in ELISA plates. (**b**) Percentage of residual binding to antibodies PG16, 10-1074, 447-52D, and 35022 after different storage times and temperatures. In cases where statistically significant differences were found, *p*-values are represented as: * *p* < 0.05; ** *p* < 0.01; **** *p* < 0.0001.

Our group has previously shown the presence of VLPs that carry HIV-1 envelope proteins by immuno-electron cryo-microscopy and immuno-NTA [4,5]. The presence of functional trimers after storage of the VLPs one month at room temperature was verified by assessing the conservation of epitopes targeted by bnAbs, including the PG16 antibody, which is specific for V1-V2-glycans and recognizes trimers in native conformation. VLPs also retained the ability to bind to the antibodies 10-1074 (V3-glycans), 447-52D (V3), and 35022 (interface) after one month at room temperature and after three months at 4 and −20 °C (Figure 2). 

Consistent with the appearance of the first evidence of particle degradation after one month at room temperature, a decrease in binding to PG16, 10-1074, 447-52D, and 35022 antibodies was observed (52.7%, 36.4%, 70.4%, and 65.2% reduction, respectively). VLPs demonstrated greater stability when stored at 4 °C. At this temperature, binding did not decrease for 35022 antibodies and decreased only by 20.0%, 20.1%, 23.9% for PG16, 10-1074, and 447-52D, respectively, after three months of storage (Figure 2). These results indicate that, although no signs of degradation in the shape of the VLPs were observed after three months at 4 °C, trimers begin to show the first evidence of degradation that does not affect all the epitopes analyzed equally. After three months of storage at −20 °C, no evidence of particle degradation was observed (Figure 1 and Figure 2). 

A possible explanation for the loss of certain epitopes with no evidence of VLP degradation would be the effect of gp120 shedding [8]. Due to this phenomenon, vaccine prototypes mimicking the HIV-1 Env trimer have incorporated several modifications in order to prevent gp120 shedding. Considering that the envelope selected for this study, AC10, does not incorporate any of these modifications, the loss of certain epitopes could be attributed to gp120 shedding. The effect of different long-term storage temperatures on gp120 shedding needs to be further investigated, in particular, if modifications were to be introduced to prevent shedding. In fact, we are currently investigating ways to improve the thermal stability of our prototypes by incorporating modifications that other groups have previously used to prevent gp120 dissociation. The two strategies that have been used for this purpose and on which we are working are: (i) The replacement of the furin cleavage site by a flexible linker [9] and (ii) The introduction of a disulfide bridge between gp120 and gp41 (A501C/T605C) in combination with the I559P substitution in gp41 [10,11].

## 4. Conclusions

We have developed a vaccine prototype based on VLP-formulated HIV-1 envelopes that have proven to be immunogenic, although the antibodies generated did not show neutralization capacity. Still, this vaccine prototype may be able to induce neutralization through multiple booster vaccinations over an extensive period of time or induce non-neutralizing antibodies with specific effector functions that have been linked to HIV control [12].

Data from the present study indicate that a vaccine prototype based on VLP-formulated HIV-1 envelopes was highly stable, preserved functional trimers and epitopes associated with the induction of broadly neutralizing responses for up to seven days at room temperature. Moreover, these epitopes remain detectable, despite having lost much of their binding capacity, even after a month of storage at room temperature. Our results thus demonstrate the potential of vaccine prototypes based on VLP formulated HIV-1 envelopes facilitating their administration globally in order to reach even regions with limited cold chain access.

## Data Availability

Not applicable.
